# Multi-Objective Optimizations of Biodegradable Polymer Stent Structure and Stent Microinjection Molding Process

**DOI:** 10.3390/polym9010020

**Published:** 2017-01-17

**Authors:** Hongxia Li, Xinyu Wang, Yunbo Wei, Tao Liu, Junfeng Gu, Zheng Li, Minjie Wang, Danyang Zhao, Aike Qiao, Yahua Liu

**Affiliations:** 1 School of Mechanical Engineering, Dalian University of Technology, Dalian 116023, China; seringwang@dlut.edu.cn (X.W.); weiyunbo@mail.dlut.edu.cn (Y.W.); tliu@mail.dlut.edu.cn (T.L.); mjwang@dlut.edu.cn (M.W.); danyangz@163.com (D.Z.); yahualiu@dlut.edu.cn (Y.L.); 2 Department of Engineering Mechanics, State Key Laboratory of Structural Analysis for Industrial Equipment, Dalian University of Technology, Dalian 116023, China; jfgu@dlut.edu.cn (J.G.); lizheng@dlut.edu.cn (Z.L.); 3 College of Life Science and Bioengineering, Beijing University of Technology, Beijing 100124, China; qak@bjut.edu.cn

**Keywords:** biodegradable polymer, stent, expansion performance, injection molding, kriging, multi-objective optimization

## Abstract

Biodegradable stents made of poly-l-lactic acid (PLLA) have a promising prospect thanks to high biocompatibility and a favorable biodegradation period. However, due to the low stiffness of PLLA, polymeric stents have a lower radial stiffness and larger foreshortening. Furthermore, a stent is a tiny meshed tube, hence, it is difficult to make a polymeric stent. In the present study, a finite element analysis-based optimization method combined with Kriging surrogate modeling is firstly proposed to optimize the stent structure and stent microinjection molding process, so as to improve the stent mechanical properties and microinjection molding quality, respectively. The Kriging surrogate models are constructed to formulate the approximate mathematical relationships between the design variables and design objectives. Expected improvement is employed to balance local and global search to find the global optimal design. As an example, the polymeric ART18Z stent was investigated. The mechanical properties of stent expansion in a stenotic artery and the molding quality were improved after optimization. Numerical results demonstrate the proposed optimization method can be used for the computationally measurable optimality of stent structure design and stent microinjection molding process.

## 1. Introduction

A stent is a tiny mesh tube used to treat arterial occlusive diseases, with an angioplasty procedure performed to partially open up the blocked vessel. After being positioned in a stenosis segment, the stent is expanded radially under the action of balloon expansion. Once the balloon is deflated and removed, the expanded stent retains its diameter and provides a scaffoldlike support structure to maintain the patency of the vessel, thereby promoting blood flow. There are three development stages of stents: bare-metal stents (BMS), drug-eluting stents (DES), and bioresorbable stents. Currently, bioresorbable stents, which can address the concerns raised by permanent metallic stents (e.g., long-term safety), is considered to be the fourth revolution in interventional cardiology [1]. Among them, biodegradable stents made of poly-l-lactic acid (PLLA) have a promising prospect thanks to high biocompatibility and a favorable biodegradation period.

Although the previous studies have shown encouraging development prospects of biodegradable polymer stents, the low stiffness of polymers and the lack of a precise and efficient method of manufacture potentially limit the development of polymeric stents. Due to the low stiffness of polymers, polymeric stents have low radial strengths compared to metallic stents with similar dimensions [2]. The deficiency of mechanical strength of polymeric stents can result in radial recoil after the deflation of balloon, which can reduce the luminal area of the vessel and, therefore, influence blood flow. In order to increase the stiffness of the polymeric stents, all currently known polymeric stents have thicker and wider struts than metallic stents. For example, the bioresorbable vascular scaffold (BVS)-B stent has a strut thickness of 0.15 mm, and both the Igaki-Tamai stent and ART18Z stent have the same thickness of 0.17 mm. However, a wider strut results in a larger surface area of stent, which may lead to higher rates of restenosis [2,3]. Moreover, stents with thicker struts can reduce stent flexibility and significantly occupy the lumen of the artery. This results in a difficult delivery of the stent and a reduction of blood flow through the lumen [2]. Furthermore, there is a significant foreshortening in the process of stent expansion. This not only affects the position of stent, but also causes injury to the vessel wall. Therefore, it is necessary to design the structure of stent to improve its mechanical properties before manufacture.

Currently, the design of polymeric stents has learnt from the design of permanent metallic stents. Among them, there are numbers of computational-based studies of parametric comparison. For example, the expansion behavior of several stents with different geometries was compared in terms of dogboning, foreshortening, elastic recoil, and so on. Migliavacca et al. [4,5] and Beule et al. [6] assessed the mechanical properties and behavior of balloon expandable stents to determine how the finite element analysis (FEA) method could be used to optimize stent designs. It is easy to perform and study the effective factors using these types of study. Although the results obtained are helpful to stent design, it is difficult to find the globally optimal design of a stent in design space.

In terms of processing methods of a polymeric stent, the traditional method of making a stent is laser-cutting, except for some coil stents with special structures. In 2008, Clarke et al. [7] proposed a method of making a polymeric stent by a microinjection molding process with a central core pin and a plurality of slides. Using the microinjection molding process to make a stent has many advantages, such as less expensive tooling, reduced part cost, more accurate parts, very fast cycles, and obtaining parts with complex geometries. However, the injection molding process has not been used commonly in stent manufacturing because of the difficulties that appear during the process of microinjecting polymeric materials, such as difficult flow, mold cavity filling conditions, as well as some difficulties in their extraction from the cavity. In addition, the warpage induced during the injection molding process has an important influence on the quality of injection molded products [8,9]. It is obvious that the injection molding parameter—including clamping force, shot size, injection velocity, packing pressure, and temperature—will affect the quality of molded parts. Therefore, the optimization of injection molding parameters will help to improve the quality of the stent.

For the optimizations, whether structural optimization of the stent or process optimization of stent microinjection, the functional relationships between design variables and design objectives are complex, nonlinear, implicit, and multimodal. Moreover, the number of function evaluations is severely limited by time. It is hard to find a global optimal design using traditional optimization algorithms, such as gradient-based algorithms. Fortunately, surrogate modeling, predominantly involving the method of Kriging [10], can address such engineering problems effectively. It can construct an approximate functional relationship between design objectives (the output) and design variables (the input), thereby replacing complex engineering computation to greatly reduce computational cost. Timmins et al. [11] adopted Lagrange interpolating polynomials (LIPs) to optimize the stent; Shen et al. [12] improved the stent’s resistance against compression and decreased internal pressure in an expanding stent by employing the artificial neural networks (ANN). Li et al. [13,14] proposed an adaptive optimization method based on Kriging surrogate model to (1) optimize the stent structure to eliminate the dogboning phenomenon during the stent expansion process and (2) optimize stent coating to prolong the effective period of drug release. Kriging surrogate model, a semi-parameter interpolation technique, is more precise and flexible compared to Lagrange interpolating polynomials and ANN, and thus widely used in multidisciplinary design optimization (MDO).

In the present paper, both the structure optimization of a biodegradable polymer stent and process optimization of polymeric stent microinjection molding were sequentially studied using an adaptive optimization method based on Kriging surrogate modeling. A multi-objective optimization was proposed to optimize the geometries of the biodegradable polymer stent so as to improve stent expansion performance. Then, microinjection molding process optimization of the optimal stent obtained from structure optimization was proposed to optimize the parameters of injection molding in order to reduce the deformation of stent, so as to improve the stent quality. The Kriging model was used to build the relationship between measures of stent expansion performance and stent geometries in the structure optimization, as well as the relationship between deformation of the stent and injection molding parameters in the microinjection molding process optimization, thereby replacing the expensive finite element reanalysis of the stent expansion and injection molding process. The optimization iterations are based on the approximate relationships for reducing the high computational cost. Optimal Latin hypercube sampling method [15] (Optimal LHS) was used to generate the initial training sample points. In the adaptive optimization process, the expected improvement (EI) function was adopted to balance local and global searches, as it tends to find the find the global optimal design, even with a small sample size. Finite element method (FEM) was used to simulate the stent expansion and stent injection molding. The numerical results and design optimization method of this work could potentially be useful in further optimization studies and development of biodegradable polymer stents, as well as development of the method of making a polymeric stent by means of the microinjection molding process.

## 2. Materials and Methods

**Figure 1:**
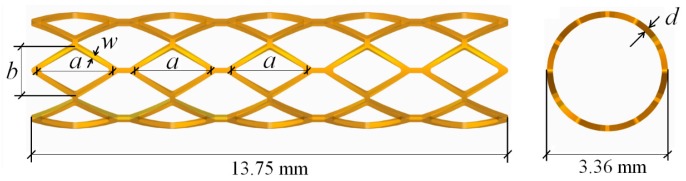
Generic polymeric stent with straight bridges based on the ART18Z platform.

Most of the currently known biodegradable polymer stents are constructed of PLLA, which has high biocompatibility and a favorable biodegradation period in coronary arteries. As an example, the bioabsorbable ART18Z stent was studied, which has a length of 13.75 mm, a thickness of 0.17 mm, and an outer diameter of 3.36 mm. The CAD model of a generic polymer stent with straight bridges based on the ART18Z platform is shown in Figure 1.

When undertaking an FEM-based design optimization, whether it is structure optimization or molding process optimization, the design optimization process consists of the following three parts:

(i) optimization problem (including design objectives, design variables, and constraints);
(ii) finite element method; and
(iii) optimization algorithm.

### 2.1. Optimization Problems

#### 2.1.1. Stent Structure Optimization

Generally, due to the low stiffness of PLLA, polymeric stents have lower radial stiffness. Hence, there is radial recoil of the stent after the deflation of the balloon. This can reduce the blood flow area though the lumen. Moreover, because of the large deformation of the stent in the expansion process, there is a significant foreshortening of the stent, which can affect not only the stent position, but also cause mechanical damage to the vessel wall. Furthermore, the larger coverage can cause neointimal hyperplasia in the vessel wall, and usually it is not more than 20%. Therefore, the multi-objective optimization design of the stent structure can be defined as:

(1) $$\begin{array}{ll} \min & \mathrm{radial}\;\mathrm{recoil},\;\mathrm{foreshortening}\\ s.t. & {\underline{x}}_{1}\leq x_{1}\leq {\bar{x}}_{1}\\ & \mathrm{coverage}\leq 20\% \end{array}$$

where $x_{1}$ is a vector of design variables of stent, ${\underline{x}}_{1}$ and ${\bar{x}}_{1}$ are the lower and upper limits of $x_{1}$, and

(2) $$\begin{array}{l} \mathrm{radial}\;\mathrm{recoil}\;(RR)\;=\frac{R_{\mathrm{loading}}-R_{\mathrm{unloading}}}{R_{\mathrm{loading}}}\times 100\%\\ \mathrm{foreshortening}\;(FS)\;=\frac{L-L_{\mathrm{unloading}}}{L}\times 100\%\\ \mathrm{coverage}\;=\frac{A_{\mathrm{stent}}}{A_{0}}\times 100\% \end{array}$$

where $R_{\mathrm{loading}}$ is the radial of the full expanded stent, $R_{\mathrm{unloading}}$ is the diameter of stent after unloading, L is the initial length of stent, and $L_{\mathrm{unloading}}$ is the length of stent after unloading. $A_{\mathrm{stent}}$ is the area of stent outer surface and $A_{0}$ is the area of a cylinder with the same diameter as the stent.

Both RR and FS are two important performance of stent expansion. However, since RR and FS of a polymeric stent are very different and have different scales, it is not easy to choose appropriate weights. If we scale both of them to [0, 1], then we might be able to assign an intermediate weight of 0.5. Therefore, the optimization problem defined in Equation (1) can be written as:

(3) $$\begin{array}{ll} \min & f(x_{1})=0.5\frac{RR-RR_{\min}}{RR_{\max}-RR_{\min}}+0.5\frac{FS-FS_{\min}}{FS_{\max}-FS_{\min}}\\ s.t. & 2.2\;\mathrm{mm}\leq a\leq 2.6\;\mathrm{mm}\\ & 1.4\;\mathrm{mm}\leq b\leq 1.6\;\mathrm{mm}\\ & 0.1\;\mathrm{mm}\leq w\leq 0.13\;\mathrm{mm}\\ & 0.1\;\mathrm{mm}\leq d\leq 0.15\;\mathrm{mm}\\ & \mathrm{coverage}\leq 20\% \end{array}$$

where $RR_{\min}$ and $RR_{\max}$ are the minimum and maximum of RR in the samples, respectively, and $FS_{\min}$ and $FS_{\max}$ are the minimum and maximum of FS in the samples, respectively. $x_{1}$ is a vector of design variables, which consists of the geometrical parameters a, b, w, and d of the stent, shown in Figure 1, in which a and b are the length and width of the diamond hole, and w and d are the width and thickness of struts.

#### 2.1.2. Stent Injection Molding Process Optimization

The warpage of a biodegradable polymer stent during the microinjection molding process is an important measure to evaluate stent molding quality. Hence, the optimization of the stent injection molding process to improve stent quality can be defined as:

(4) $$\begin{array}{ll} \min & \mathrm{Warpage}\\ s.t. & 80\;{}^{\circ}\text{C}\leq T_{\mathrm{mold}}\leq 90\;{}^{\circ}\text{C}\\ & 190\;{}^{\circ}\text{C}\leq T_{\mathrm{melt}}\leq 205\;{}^{\circ}\text{C}\\ & 0.13\;{\mathrm{cm}}^{3}/\text{s}\leq v\leq 0.25\;{\mathrm{cm}}^{3}/\text{s}\\ & 75\%\leq P\leq 90\%\\ & 0.1\;s\leq t\leq 2\;s \end{array}$$

where $T_{\mathrm{melt}}$ and $T_{\mathrm{mold}}$ are the temperature of the melt and the mold, respectively; v is the flow rate, P is the packing pressure, and t is the packing time.

### 2.2. Finite Element Methods

#### 2.2.1. Stent Expansion

**Figure 2:**
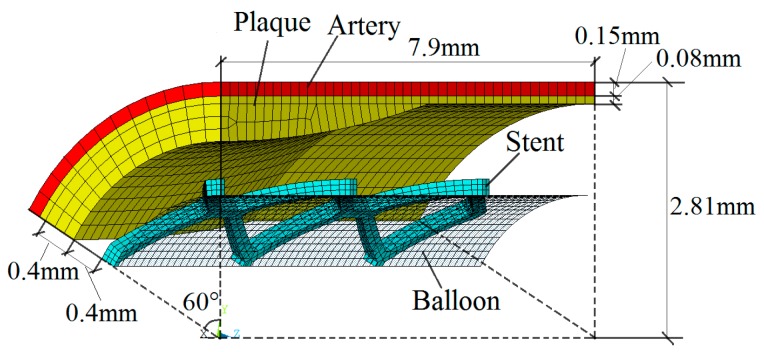
The finite element model of polymeric stent expansion in a stenotic artery. The 8-node solid element was assumed for the artery, plaque, and polymeric stent, while the shell element was assumed for the balloon. The balloon consists of 896 elements, with 56 elements along its length and 16 elements in circumference. The polymeric stent was discretized by 930 elements with 3 elements in the modeled thickness. The artery consists of 504 elements and the plaque consists of 960 elements.

There are many FEMs used to investigate the expansion process of stents in the published studies [16,17,18]. Among them, a three-dimensional simulation model combining artery, plaque, stent, and balloon is thought to be realistic and available. The finite element model in three-dimensions is shown in Figure 2. ANSYS 15.0 (ANSYS, Inc., Canonsburg, PA, USA) program was used to simulate the polymeric stent expansion in stenotic artery. Because of the symmetry of the entire model, 1/12 of the entire model—with 1/6 in the circumferential direction and 1/2 in the longitudinal direction—was simulated in this study. The balloon was placed inside the polymeric stent. The plaque was not in contact with the polymeric stent at the beginning of expansion. The thicknesses of plaque were modeled to be 0.48 mm at proximal and 0.08 mm at distal. The artery was modeled to have an identical thickness of 0.15 mm. The balloon was modeled with a length of 7.75 mm and a thickness of 0.05 mm.

The contact between balloon and stent and the contact between stent and plaque were considered, while the friction between them was ignored. A radial displacement with two steps was applied to the balloon. In the first step, the outer diameter of stent was expanded to the inner diameter of the artery (5.32 mm in this study). Then, the balloon was deflated to the initial state in the second step. The symmetry constraints were applied to the symmetry parts of artery, plaque, stent, and balloon, while the distal parts of them were free.

**Figure 3:**
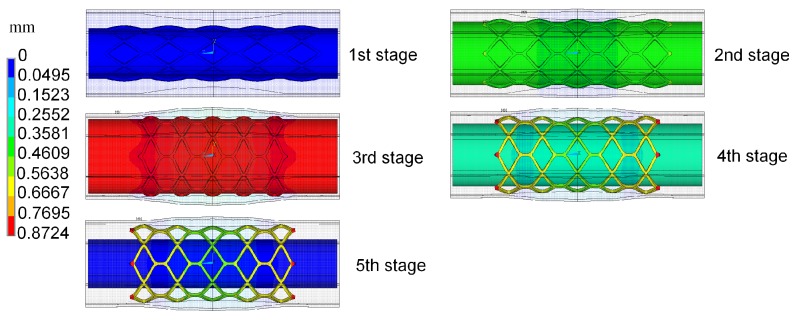
The expansion process of a polymeric stent in a stenotic artery displayed in symmetry.

In this simulation, the material properties were based on the data available from previous studies [19]. Therein, the PLLA stent had an elastic modulus of 3.363 GPa, a Poisson ratio of 0.45, and a yield stress of 40 MPa. A linear isotropic and nearly incompressible model was assumed for the plaque and artery with elastic moduli 1.75 and 2.19 MPa, as well as a Poisson ratio of 0.499. The balloon was modeled as a Mooney–Rivlin hyperelastic shell with two parameters: $c_{01}=1.0688$ MPa, $c_{01}=0.71092$ MPa. The polymeric stent expansion process in the stenotic artery is shown in Figure 3. The stent expands as the balloon is inflated (see 1st to 5th stage). After the deflation of balloon, the stent remains the shape to form a vascular support (see 4th and 5th stages).

#### 2.2.2. Stent Microinjection Molding

**Figure 4:**
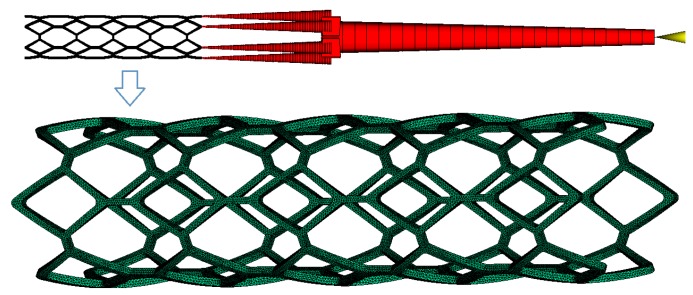
The simulation model of stent injection molding based on the ART18Z platform.

FEM was also used in the injection molding process optimization. Xu et al. [8] firstly proposed an integrated FEM of the injection molding process to optimize process parameters using a neural network. In this study, there are six repeating cells in the circumference of the ART18Z stent. In order to balance the melt flow in the cavities, six gates were arranged at the distal ends of the stent. The simulation model of stent injection molding based on the ART18Z platform is shown in Figure 4. Moldflow program was used to simulate the filling process of the polymeric stent. The stent finite element model consists of 80,670 elements with 40,227 nodes.

**Figure 5:**
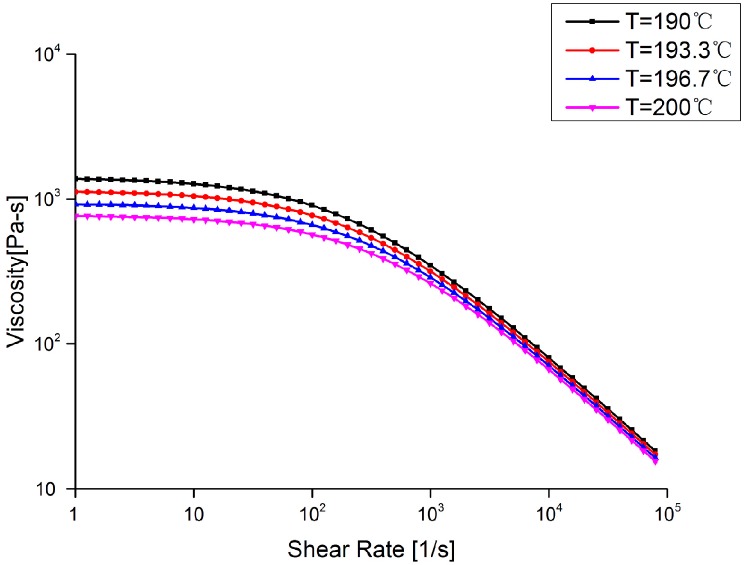
The rheological properties of poly-l-lactic acid (PLLA) with crystalline melt mass-flow rate (MFR) of 0.55 g/min measured under the conditions of a temperature of 200 °C and a load of 5 kg.

**Figure 6:**
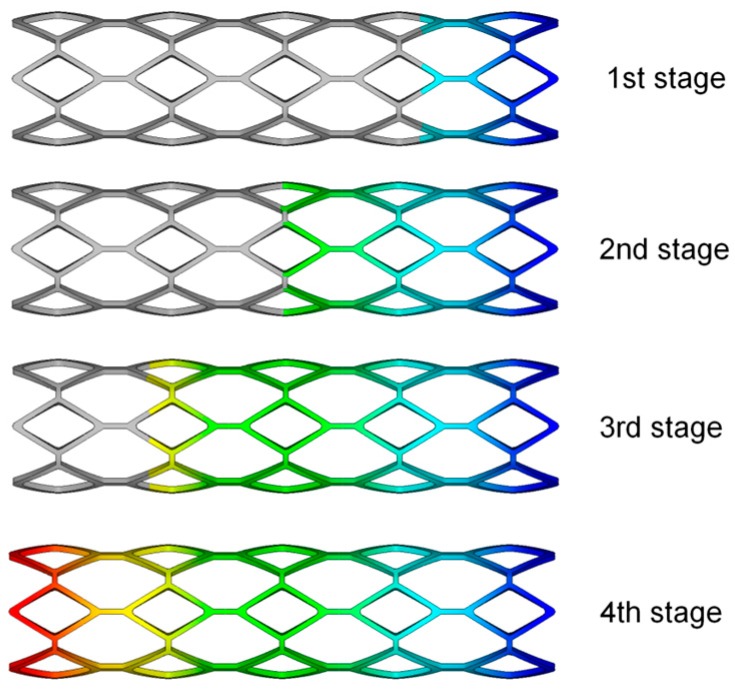
The injection molding process of a stent based on the ART18Z platform.

In this simulation, the material of the polymeric stent was considered as a crystalline structure with a melt mass-flow rate (MFR) of 0.55 g/min, measured under the conditions of a temperature of 200 °C and a load of 5 kg. From PLLA’s rheological properties (shown in Figure 5), it can be seen that viscosity of the PLLA decreases slightly by increasing shear rate from 1 to 1 × 10^2^ s^−1^, and the effect of temperature on viscosity is remarkable. From 1 × 10^2^ to 1 × 10^5^ s^−1^, the viscosity will decrease rapidly, and not be obviously influenced by temperature. The injection molding process of the stent is shown in Figure 6. The melt flows into the cavity from the right distal end, and then fills the entire cavity. The maximum warpage is distributed at the left distal end of stent, while the minimum warpage is located at the proximal end of stent.

### 2.3. Optimization Algorithm

In engineering optimization design, when the number of function evaluations is limited by time or cost, it is appropriate to address this problem by surrogate models, especially Kriging surrogate model. The key steps in surrogated model-based optimization process are:

(i) define an optimization problem (the design variables (or inputs), measures of performance (or outputs), upper and lower bounds);
(ii) get the initial training samples by a space-filling algorithm (or design of experiment), and then analyze each design to get the objective function value;
(iii) construct the approximate relationship between design objective and design variables based on the training samples;
(iv) choose an optimization algorithm to obtain the optimal design based on approximate function; and
(v) check the convergence criteria.

**Figure 7:**
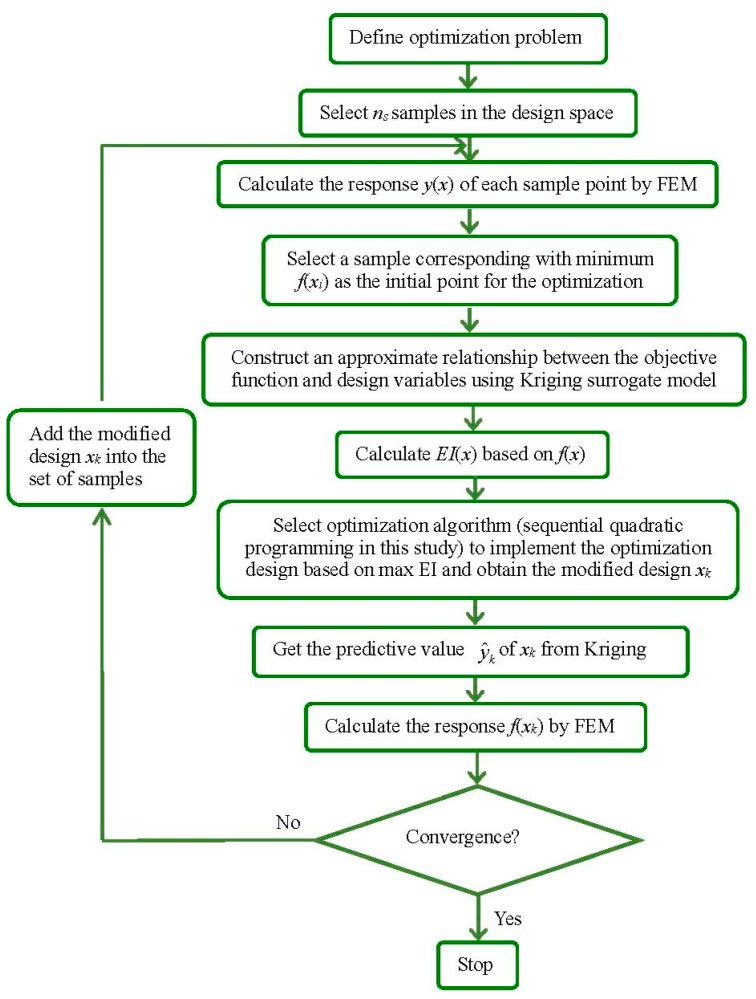
Flowchart showing the process of optimization algorithm based on surrogate model.

Figure 7 depicts the process of an optimization algorithm based on the surrogate model. In the present study, altered adaptive optimization method based on Kriging surrogate model is employed to minimize the design objectives. Kriging surrogate model [10,20] coupled with a design of experiments (DOE) algorithm [21] is used to create approximate functional relationship between design objective and design variables. The basic idea of Kriging is to predict the value of a function at a given point by computing a weighted average of the known values of the function in the neighborhood of the point. It derives the best linear unbiased estimator, based on assumptions on covariance, makes use of Gauss–Markov theorem to prove independence of the estimate and error, and employs very similar formulae. A new value can be predicted at any new spatial location by combining the Gaussian prior with a Gaussian likelihood function for each of the observed values [22]. As a semi-parametric approach, Kriging model is more flexible in application than interpolation method, which involves a parametric model, and is more powerful in making global prediction than semi-parametric model [23]. Optimal Latin hypercube sampling (LHS) and orthogonal LHS are adopted to select sample points in the design space of the stent’s geometries and in the design space of the stent injection molding process, respectively. Expected improvement (EI) function [21] is adopted to balance the local and global search so as to find the optimal result. The optimization iteration starts from a sample point corresponding with the minimum objective in training samples. We modify the Kriging model in each iteration step until the error between Kriging predictive value and FEM simulation falls below a given tolerance. The optimization process stops when the following conditions of convergence are met:

(5) $$\begin{array}{l} \frac{EI_{k}}{Y_{\max}-Y_{\min}}\leq \varepsilon_{1}\\ \left|f_{k}-{\hat{y}}_{k}\right|\leq \varepsilon_{2}\\ \left|f_{k}-f_{k-1}\right|\leq \varepsilon_{3} \end{array}$$

where $EI_{k}$ denotes the functional value of *EI* at the *k*_th_ iteration. $Y_{\max}$ and $Y_{\min}$ are the maximum and minimum responses, respectively, among the sample points. $f_{k}$ and $f_{k-1}$ are the values of objective functions at the $f_{k}$th and $f_{k-1}$th iteration, respectively. ${\hat{y}}_{k}$ denotes the predicted value of Kriging at the kth step.

## 3. Results

### 3.1. Results of Stent Structure Optimization

The radial recoil and foreshortening based on ART18Z stent platform was minimized by the proposed method, with the constraint that coverage is less than 20%. The initial trial samples, including an initial experience design and another 30 samples generated by optimal LHS, were selected for constructing Kriging surrogate model. The optimization iteration is based on the constructed surrogate model. The polymeric stent expansion processes in the stenotic artery for all trial samples are simulated by ANSYS software. EI function was employed to balance local and global search in the design space. The optimization process started from the initial point with the minimum value of optimization function among all the sample points. Twenty-one iterations were needed to obtain the optimal solution.

The optimization result was compared to the original design, as well as to the comparable stent, which has the same geometries as the original stent except for the width and thickness of struts, as shown in Table 1. With the comparison to the original stent, the optimal stent has similar mechanical properties as the original stent, even though both the width and thickness of the struts of the optimal stent were decreased by 0.02 mm. The reduction of the strut width of the optimal stent is conducive to the smaller neointimal hyperplasia and better stent coverage, which can reduce the risk of in-stent restenosis. Moreover, although the radial recoil of the optimal stent is slightly larger than that of the original stent (due to the smaller thickness of the optimal stent), it has a larger lumen area than the original stent. When compared to the stent having the same geometrics as the original stent except for the width and thickness of struts (named “comparable stent” in this study), the optimal stent has lower radial recoil and larger lumen area, although both of them have the same width and thickness of struts.

The radial recoil and foreshortening are two conflicting objectives. Generally, the foreshortening is greater when the stent diameter expands to a larger degree. However, a larger deformation of the polymeric stent results in a larger region of greater plastic deformation, thereby the elastic deformation and the region in which it occurred are relatively small. This results in smaller radial elastic recoil caused by the elastic deformation of stent. Furthermore, because both the radial recoil and foreshortening are not only related to the stent structure, but also connected with the materials and expansion process of the polymeric stent, it is hard, even impossible, to eliminate them.

**Figure 8:**
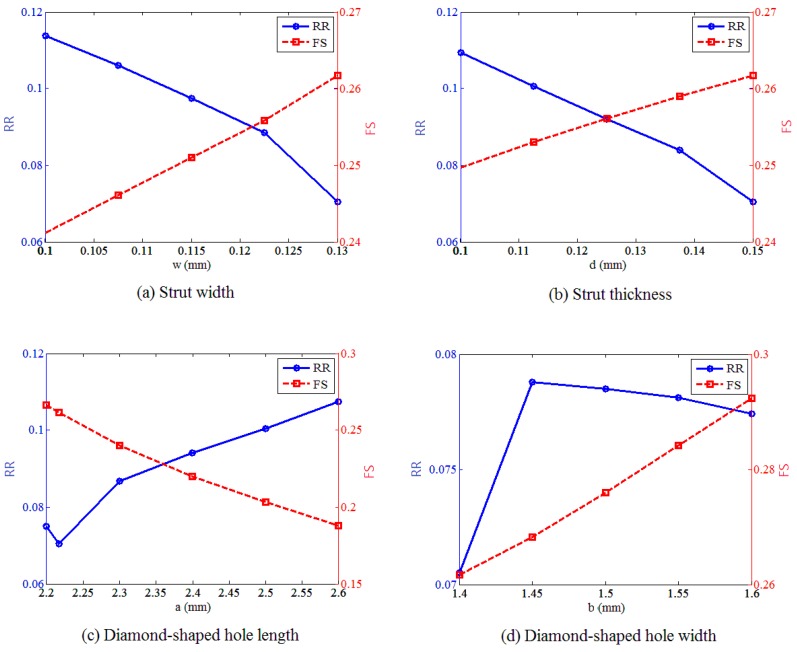
The effect of each design variable on the radial recoil and foreshortening when fixing other variables at their optimal values: (**a**) the effect of strut width on the radial recoil and foreshortening; (**b**) the effect of strut thickness on the radial recoil and foreshortening; (**c**) the effect of diamond-shaped hole length on the radial recoil and foreshortening; (**d**) the effect of diamond-shaped hole width on the radial recoil and foreshortening.

In order to study the effect of the strut dimensions on the expansion performance of stent, the effect of each design variable on the radial recoil and foreshortening were studied independently, with other variables fixed at their optimal values, as shown in Figure 8. These results demonstrated that radial recoil and foreshortening are two conflicting measures of stent performance. The stent radial recoil decreased with the strut width and strut thickness, because the stent mechanical strength increased with the increase of these two design variables. Meanwhile, the foreshortening increased with these two design variables, because the foreshortening was larger when the stent was expanded to a greater degree. There was an optimal value of the diamond-shaped hole length to minimize the stent radial recoil in the design range, while the diamond-shaped hole width had a value that corresponded to the maximum of radial recoil. This was because the stent studied consists of diamonds and hexagons, which had common edges. The geometry of the diamond affected the geometrical structure of the hexagon, which also had an impact on the stent radial recoil. The foreshortening of stent decreased with the length of the diamond-shaped hole, and increased with the width of the diamond-shaped hole.

### 3.2. Results of Stent Injection Molding Process

**Figure 9:**
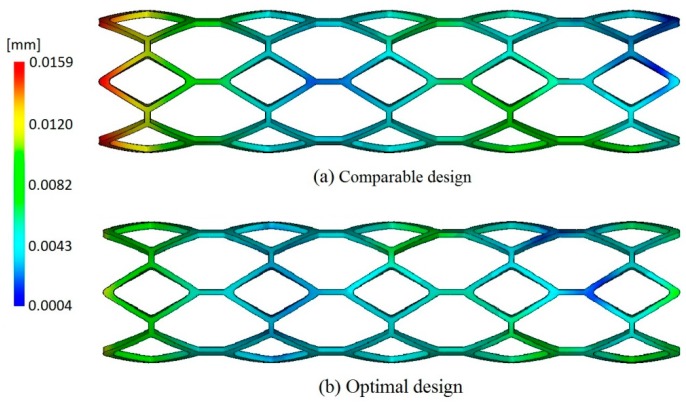
The distribution of warpage of the comparable design and optimal design.

16 initial trial samples generated by orthogonal LHS were used for constructing Kriging surrogate model to build the approximate relationship between stent warpage and design variables including temperature of the melt and the mold ($T_{\mathrm{melt}}$ and $T_{\mathrm{mold}}$), flow rate (v), packing pressure (P), and packing time (t). Twenty-one iterations were needed to obtain the optimal solution. The optimization result was compared to the best design point, which corresponding to the minimum value of optimization function among all the initial trial sample points, as shown in Table 2. The warpage of the optimal design was reduced by 28.3%, which was helpful to improve the molding quality of stent. The distribution of warpage of the comparable design corresponding to the best design among trial samples and optimal design are shown in Figure 9, from which it can be observed that the optimal design has smaller deformation than the comparable design.

**Figure 10:**
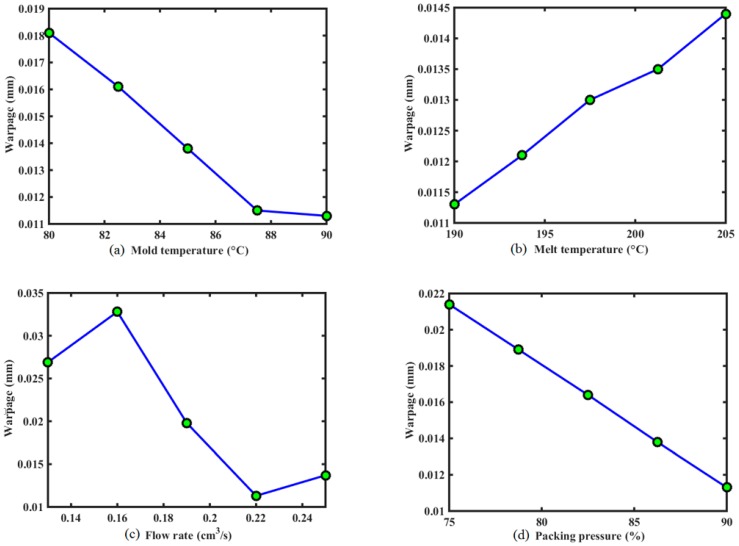

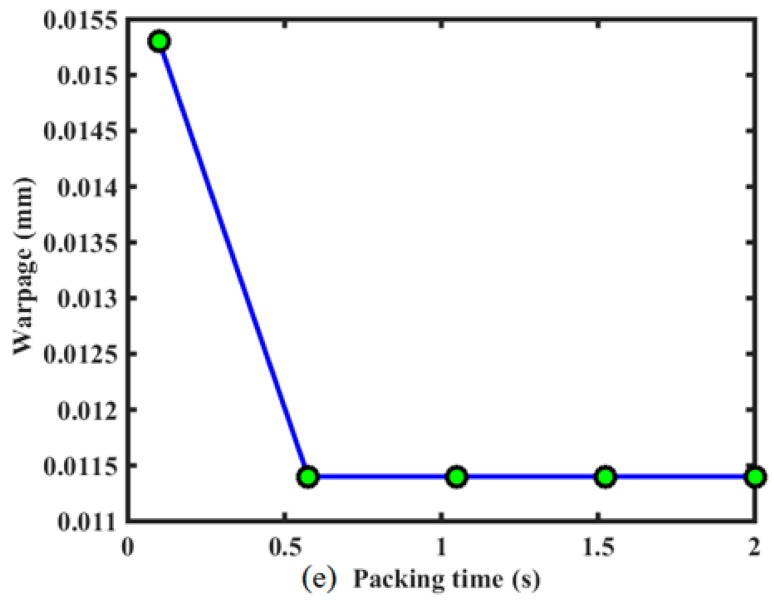
The effect of each design variable on the warpage, when fixing other variables at their optimal values: (**a**) the effect of mold temperature on the warpage of stent; (**b**) the effect of melt temperature on the warpage of stent; (**c**) the effect of flow rate on the warpage of stent; (**d**) the effect of packing pressure on the warpage of stent; (**e**) the effect of packing time on the warpage of stent.

In order to study the effect of injection molding process parameters on the warpage of the stent, the computation of the single factor analysis is performed by fixing other factors in the optimal solution, the results of which are plotted in Figure 10. It reveals that, in the given design domain, these injection process parameters do not contribute equally to the warpage of the stent. The flow rate and packing pressure have the most substantial impact, followed by mold temperature, melt temperature, and packing time.

In Figure 10, the warpage decreases as the mold temperature increases. For one thing, the lower mold temperature usually causes larger shear stress near the wall, which subsequently evolves into the larger flow-induced residual stress in the part. This will result in greater warpage after ejection, finally. Besides, the higher mold temperature can increase the cooling time. If the packing process is not reasonable, it will cause greater shrinkage in the part, which results in larger warpage. Figure 10 presents the phenomenon of warpage decreasing when mold temperature changes from the lowest value to the reasonable value for the given range.

The warpage increases with the melt temperature. The effect of melt temperature is similar to that of mold temperature. Increasing melt temperature can increase the cooling time as well. Then, the shrinkage of the part relies on the packing process. If packing pressure is not large enough or packing time is not long enough, there is greater shrinkage in the part, which leads to the greater warpage.

There is a nonlinear relationship between the warpage and the flow rate. Lower flow rate will increase the viscosity due to more heat lost at the flow front, which can make shear stress increase. However, higher flow rate can avoid heat loss, but increase the shear rate, which can cause higher shear stress as well. These usually result in higher residual stress in the part, and then cause serious warpage. Actually, low flow rate means long filling time, while, on the contrary, high flow rate represents short filling time. These process conditions require high injecting pressure, which is not expected during manufacturing since it indicates more energy consumption. Therefore, the reasonable value of flow rate should be determined carefully.

The warpage decreases when the packing pressure increases. This is because the higher packing pressure could ensure injection of a certain material in order to compensate for the shrinkage during cooling. However, packing pressure cannot be too high, since it can induce over-packing, which is not beneficial to prevention of warpage. Therefore, an intermediate packing pressure is preferred.

The warpage decreased with the packing time, and then had little effect on the stent warpage, as shown in Figure 10. Because the sizes of stent and the injecting gate are both very small, the cooling time in the process is not too long. Although packing is proceeding due to hot runner and gate, no melt material can be injected into the cavity. A short packing time is sufficient, and the effect of long packing time on warpage is the same as that of this short packing time. Therefore, there exists the platform on the curve in Figure 10.

## 4. Discussion

For the biodegradable polymeric stent, the low stiffness of polymers and the lack of a precise and efficient method of manufacture are the potential limitations of the development of polymeric stents. Due to the low stiffness of polymers, polymeric stents have low radial support capacity. Generally, the polymeric stents have been designed with thicker and wider struts in order to increase their stiffness. However, the thicker struts can cause a reduction of stent flexibility, as well as a reduction of the area of blood flow through the artery lumen. A wider strut also increases the level of injury to the vessel. In the present paper, in order to decrease the thickness and width of the struts of polymeric stents, the range of the thickness and width of polymeric stents studied here were set to be [0.1, 0.15] and [0.1, 0.13]. Obviously, reducing the thickness and width of struts will decrease the stent radial stiffness, and this will cause radial recoil after the deflation of balloon. Consequently, the optimization design of stent structure has an objective to minimize the radial recoil of the stent so as to provide sufficient support for the stenotic artery, as well as an objective to minimize the foreshortening so as to reduce the level of injury to the vessel. After optimization, with both the width and thickness of the struts having been reduced by 0.02 mm, the optimal stent had similar expansion performance as the original stent.

It should be acknowledged that when the stent was implanted into a stenotic artery with different diameters, the stent expansion performance (including the radial recoil and foreshortening) will differ from that in this study, and this will lead to a different optimal result of stent structure. Similarly, the injection molding process optimization result would be different if a different stent was investigated. However, as an example, this study illustrated how to use surrogate modeling to optimize stent structure and stent injection molding process, with the aim of demonstrating that a superior structure of stent can be designed and refined computationally.

Currently, metallic stents are approaching a mature stage of evolution, while degradable polymeric stents are still in the development stage. It is urgent that polymeric stent designs learn from the design of permanent metallic stents. There have been many parametric studies of metallic stents by their comparative tests. Dumoulin and Cochelin [24] evaluated and characterized the mechanical properties and behaviors of a balloon expandable stent. In terms of stent design, Migliavacca et al. [4,5] and Beule et al. [6] assessed the mechanical properties and behavior of balloon expandable stents to determine how the FEA method could be used to optimize stent designs. These studies are beneficial to aid stent design, and it is easy to analyze the effective factors. However, it is difficult to find the globally optimal solution in the design space because the functional relationship between the geometrical parameters and dilation performance of stent is complex, nonlinear, and implicit, in addition to the complicating factors of time and cost.

In contrast to the expansive computational simulations employed in the comparison test studies, the surrogate modeling approach uses surrogate models to represent the relationship between design objectives and design variables [10]. Harewood et al. [24] optimized the radial stiffness of a single ring. Li et al. [12,14] optimized stent dogboning and drug release, respectively. Grogan et al. [25] performed an optimization for maximum radial strength. When considering multiple objectives, Pant et al. [26] constructed the Pareto fronts generated by treating each objective separately. Bressloff [2] recast the multi-objective optimization as a constrained problem. Although there have been some previous studies about surrogate model-based optimization of stents, and the potential for future studies have been demonstrated, the surrogate models, especially the Kriging surrogate model, as well as the optimization tools associated with Kriging, have not been used as efficiently as they could have been. Currently, the existing research of multi-objective optimization of the stent just focuses on a single or a few objectives. However, the multi-objective optimization of stents design involves a large number of design objectives, especially for the biodegradable stents, in which the degradation should be considered. Therefore, in future research, the optimization of biodegradable stents should consider more design objectives using the surrogate model-based optimization method combined with effective optimization tools.

In terms of stent manufacture, as a new method of making a polymeric stent, microinjection molding has demonstrated its potential for the future, although it has not been widely used in production. Because the stent has a complex spatial structure and tiny struts, it is difficult to make it by injection molding. The parametric study of the stent injection molding process can provide guidance for the manufacturing of it. Moreover, the wall shear stress of the stent in the injection molding process results in the safety of stent. In addition, the residual stress of the stent in the injection molding has an influence on the service performance of stent. Furthermore, the strength of weld lines, as well as the overall polymer orientation, will play a major role in the mechanical strength of the stent. However, there is quite a lack of research in these areas. Hence, there are potential challenging studies of the stent injection molding process in future.

## 5. Conclusions

This article presents a finite element analysis-based design optimization method combined with Kriging surrogate model to improve the expansion performance and the microinjection molding process of a bioresorbable polymeric stent with tiny struts. The Kriging surrogate model coupled with DOE methods was adopted to construct an approximate relationship between the objective function and geometries. The EI function was employed to balance local and global searches with the aim of finding the global optimal design. The results show that the proposed optimization method could be used for both the stent structure optimization and stent microinjection molding process effectively and conveniently. This provides a new method of the structure design and injection molding process design of a polymeric stent, and represents a new direction of research. This optimization method combined with experimental verification can serve as a useful tool for stent design and injection molding process design before manufacture.

## Figures and Tables

**Table 1:** Optimization result of stent structure compared to the original stent and comparable stent.

Stents	w (mm)	d (mm)	a (mm)	b (mm)	Coverage (%)	Lumen area (mm^2^)	*RR*	*FS*
Original stent	0.15	0.17	2.4	1.56	18.97	14.4422	0.0669	0.2538
Comparable stent	0.13	0.15	2.4	1.56	16.56	13.7382	0.0963	0.2372
Optimal stent	0.13	0.15	2.2175	1.4	16.20	14.5918	0.0705	0.2617

*RR*: radial recoil; *FS*: foreshortening.

**Table 2:** Optimization result of stent injection molding process compared to the design point with the minimum value of optimization function among all the initial trial sample points (named comparable design).

Stents	$T_{\mathrm{mold}}$ (°C)	$T_{\mathrm{melt}}$ (°C)	v (cm^3^/s)	P (%)	t (s)	Warpage (mm)
Comparable design	88.9	193.0	0.2	83.9	1.9	0.0159
Optimal design	90	190	0.2	90	1.3	0.0114
